# Cross-Species Integrative Functional Genomics in GeneWeaver Reveals a Role for *Pafah1b1* in Altered Response to Alcohol

**DOI:** 10.3389/fnbeh.2016.00001

**Published:** 2016-01-21

**Authors:** Jason A. Bubier, Troy D. Wilcox, Jeremy J. Jay, Michael A. Langston, Erich J. Baker, Elissa J. Chesler

**Affiliations:** ^1^The Jackson LaboratoryBar Harbor, ME, USA; ^2^Department of Bioinformatics and Genomics, The University of North Carolina at Charlotte, North Carolina Research CampusKannapolis, NC, USA; ^3^Department of Electrical Engineering and Computer Science, University of TennesseeKnoxville, TN, USA; ^4^School of Engineering and Department of Computer Science, Baylor UniversityWaco, TX, USA

**Keywords:** behavioral genomics, combinatorial data integration, functional genomics

## Abstract

Identifying the biological substrates of complex neurobehavioral traits such as alcohol dependency pose a tremendous challenge given the diverse model systems and phenotypic assessments used. To address this problem we have developed a platform for integrated analysis of high-throughput or genome-wide functional genomics studies. A wealth of such data exists, but it is often found in disparate, non-computable forms. Our interactive web-based software system, Gene Weaver (http://www.geneweaver.org), couples curated results from genomic studies to graph-theoretical tools for combinatorial analysis. Using this system we identified a gene underlying multiple alcohol-related phenotypes in four species. A search of over 60,000 gene sets in GeneWeaver's database revealed alcohol-related experimental results including genes identified in mouse genetic mapping studies, alcohol selected *Drosophila* lines, *Rattus* differential expression, and human alcoholic brains. We identified highly connected genes and compared these to genes currently annotated to alcohol-related behaviors and processes. The most highly connected gene not annotated to alcohol was *Pafah1b1.* Experimental validation using a *Pafah1b1* conditional knock-out mouse confirmed that this gene is associated with an increased preference for alcohol and an altered thermoregulatory response to alcohol. Although this gene has not been previously implicated in alcohol-related behaviors, its function in various neural mechanisms makes a role in alcohol-related phenomena plausible. By making diverse cross-species functional genomics data readily computable, we were able to identify and confirm a novel alcohol-related gene that may have implications for alcohol use disorders and other effects of alcohol.

## Introduction

With the expanding diversity of sequenced genomes and decreasing costs of functional genomic experiments, numerous large-scale genomic, transcriptomic, metabolomics, and proteomic studies have been performed. Public repositories and private data collections of functional genomic data provide a rich context for the discovery of pathways underlying biological processes conserved across species and experimental systems. Exploiting these data for discovery of disease related genes requires extensive harmonization of identifiers for biological molecules and scalable, integrative analysis strategies for aggregating and synthesizing data across diverse technologies, species, and experimental paradigms. To address this need, we developed a publicly available web-based software system, GeneWeaver.org, that combines curated and user submitted functional genomic data (i.e., gene sets consisting of genes and gene products) from diverse experiments across numerous species, harmonized through homology and identifier translation tables, and coupled to a suite of software tools for combinatorial integration and analysis of these diverse data (Baker et al., 2012). The software in GeneWeaver includes analysis tools for evaluation of gene set—gene set intersections and scalable graph algorithms for set-set matching to prioritize genes and gene sets of interest. These tools are directly incorporated into GeneWeaver, allowing users to prioritize genes in real time based on the relations among user-selected and user-entered gene sets. This strategy enables users to find consensus among highly heterogeneous experiments implicating genes in disease.

To demonstrate the application of integrative analysis of genome-wide functional genomics experiments, we applied GeneWeaver to identify novel genes related to alcohol-use disorder. Alcohol-use disorders are a major public health problem; over 16 million US adults had an alcohol-use disorder in 2013 (SAMHSA, 2013), and globally alcohol misuse is the leading risk factor for premature death and disability among people ages 15 and 49 (Lim et al., 2012). Unfortunately, efforts to identify the causative biological and genetic factors, and thus to develop novel pharmacological therapies, are hindered by the complexity of the disorder. Many molecular entities encoded throughout the genome underlie the various facets of alcohol-use disorder. Investigators have used a variety of species and experimental strategies to identify behavioral and molecular responses to alcohol. Conserved pathways or orthologous genes have been implicated in alcohol-related phenotypes across species (Morozova et al., 2007; McGary et al., 2010). Each study has advantages afforded by specific strengths of the individual disease models and the genetic tools available to interrogate these models, each representing various facets of alcohol effects and the progression to alcohol-use disorders. Finding consensus among these results has the potential to reveal the central mechanisms of disease, dissociated from experimental idiosyncrasies. The tremendous breadth of biological data, diversity of models, and the complexity of matching multiple genes to multiple phenomena across species has made the discovery of convergent findings difficult. Furthermore, many of the genome-wide platforms only probe a susbest of biomolcular entities. In GeneWeaver analyses, we account for the lack of comprehensive coverage in diverse functional genomic platforms by evaluating only those features in the possible intersection of each pair of data sets. In the present study, we apply our novel approach that incorporates genome-wide functional genomics experiments to aggregate convergent evidence for the discovery of novel gene-alcohol-use disorder associations. Importantly, this approach has wide applicability for finding genes in many other disease areas.

Data from GeneWeaver were queried to find alcohol related studies and analyzed to identify highly connected genes, i.e., those found in multiple experimentally associated alcohol-relevant gene sets. These results were compared to rigorously curated and often widely studied genes from Online Mendelian Inheritance in Man (OMIM) (Amberger et al., 2015) that were previously associated with alcohol-use disorder in humans or to Mammalian Phenotype (MP) Ontology term (Smith and Eppig, 2009) annotations to alcohol-related phenotypes in mice (Bubier and Chesler, 2012). A single gene, *Pafah1b1*, was determined to be the most highly connected and was not yet previously associated with alcohol use disorder in the annotated resources. Utilizing an existing viable heterozygous knock-out in mice we validated a role for *Pafah1b1* in several alcohol-related phenotypes. These results demonstrate the potential of integrative genomics to identify novel candidate genes for human diseases.

## Materials and methods

### Integrative genomics in GeneWeaver.Org

#### Database

GeneWeaver's database currently contains ~75,000 gene sets. Data have been curated as described in Baker et al. (2012). Briefly, each gene set is assigned a Tier. Tiers I, II, and III represent public resources, machine generated resources, and human curated data sets, respectively. Tiers IV and V represent data submissions from users that are either pending curatorial review or stored for private use. To find convergence of experimentally derived gene associations from genomewide experiments the query was restricted to Tier III and IV. The database was queried (Date: Aug 2011) for Tier III and IV alcohol-related gene sets from three major experiment types: (i) QTL candidate genes, (ii) GWAS candidates, and (iii) differential expression experiments. A query for “Alcohol or Alcoholism,” followed by manual review omitting false positive search results, e.g., those for which alcohol was mentioned in the publication abstract but was not relevant to the specific gene set, resulted in the retrieval of 32 data sets.

#### Hierarchical similarity graph

The Hierarchical Similarity Graph tool in GeneWeaver is used to group experimentally derived gene-set results based on the genes they contain. For a collection of input gene sets, this tool presents a graph of hierarchical relationships in which each terminal node represents individual gene sets and each parent node represents gene-gene set bicliques found among combinations of these sets using the maximal biclique enumeration algorithm (MBEA) (Zhang et al., 2014). The resulting graph structure is determined solely from the gene-set intersections of every populated combination of gene sets. In terms of gene sets, the smallest intersections (fewest gene sets, most genes) are at the right-most levels, and the largest intersections (most gene sets, fewest genes) are at the left of the graph. To prune the hierarchical similarity graph, bootstrapping is performed. The graph in the present analysis was sampled with replacement at 75% for 1000 iterations; node-node parent-child relationships occurring in greater than 50% of the results were included in the bootstrapped graph.

#### GeneSet graph

The GeneSet Graph tool generates a bipartite graph visualization of genes and gene sets. GeneWeaver operates on graphs with two sets of vertices, where genes are represented in one partite set, and gene sets represented in the other. A degree threshold is applied on the gene partite set to reduce the graph size. In the gene-set graph visualization tool, low-degree gene vertices are displayed on the left, followed by the gene-set vertices. High-degree genes are displayed on the right, in increasing order of connectivity.

#### Comparison to known alcohol-related genes

Tier I data in GeneWeaver refers to gene sets from curated data obtained from major public resources including gene annotations to Mammalian Phenotype Ontology (MP) and Gene Ontology (GO), curated functional associations in Neuroinformatics Framework (Gardner et al., 2008), and curated chemical-gene interactions in the Comparative Toxicogenomics Database (Davis et al., 2013). These data comprise a source of “ground truth” validated associations from gene to biological constructs. Resource-grade data is updated on a 6-month cycle. A search of tier I resources for canonical genes associated with alcohol resulted in 52 gene sets. These were connected with MP terms (Smith and Eppig, 2009), or the Online Mendelian Inheritance in Man (OMIM) database (Amberger et al., 2015). The Boolean Algebra tool provides gene-set combinations by deriving new sets consisting of the union, intersection, or high-degree genes within a group of gene sets, i.e., those that are found in a number of gene sets exceeding user-defined thresholds. With this feature, large numbers of gene sets can be compared or collapsed into a smaller number of gene sets. Using (Baker et al., 2012) this tool, these gene sets were all merged to make a single set of “known alcohol genes.”

#### Emphasis genes

The known alcohol genes identified using the Boolean Algebra tools were designated as “emphasis genes.” Nodes containing emphasis genes are highlighted on the Hierarchical Similarity Graph to identify those specific nodes that contain genes previously associated with human alcoholism or mouse alcohol-related phenotypes. This analysis reveals that although many genes have been identified in specific studies of alcoholism, the most highly represented genes among the 32 data sets are not currently annotated to alcoholism. The highest node contains *Pafah1b1*, which was selected for candidate gene validation in this work.

### Experimental validation of *Pafah1b1* in alcohol-related behavior

#### Mice

All work was approved by the Institutional Animal Care and Use Committee at The Jackson Laboratory (JAX). Experiments were performed during the light phase of the light:dark cycle. Mice were group-housed with pine shavings, Shepard shacks, and nesting materials. Mice were maintained in specific-pathogen free facilities with sterilized acidified (pH 2.5–3) water and NIH31 5K52 chow.

To produce the heterozygous knockout mice, the floxed allele of *Pafah1b1b* (*Pafah1b1*^*tm*2*Awb*^; JR#008002 129S-*Pafah1b1*^*tm*2*Awb*^^∕^J) (Hirotsune et al., 1998) was crossed to male mice with germline expressing Cre (Protamine 1 promoter driven Cre; JR#003328 129S/Sv-Tg(Prm-cre)58Og/J) (O'Gorman et al., 1997) mouse to produce 129S-*Pafah1b*^*tm*2.2*Awb*^/Ejc. Two cohorts of adult heterozygous mice were tested in our testing protocol; cohort 1 included 18 heterozygous mutants (eight females and 10 males) and 22 WT mice (11 females and 11 males) which were tested at 18 weeks of age, and cohort 2 comprised 16 heterozygous mutants (10 females and six males) and 13 WT mice (10 females and three males) tested at both 18 weeks of age (hypothermia 2.25 g/kg) and again at 6–8 months of age (sufficiently removed from initial testing to avoid development of chronic tolerance).

### Overall testing procedure

#### General behavioral testing procedures

Mice were subjected to a brief battery of non-invasive behavioral tests to assess activity, anxiety, and alcohol preference (Hamre et al., 2007). Mice were habituated to the testing room for 1 h prior to testing. Two experimenters participated in the testing, and a single experimenter handled the mice for each test. The same individuals were in the room during all sessions of a particular test.

#### Assessment of alcohol effects

For cohort 1, on the first day naïve mice were tested in the open field, rotarod, and light-dark box, and the baseline body temperature was taken. On day 2 the mice were injected with 1.25 g/kg EtOH before performing the same series of assays. At 60 min post-injection their body temperature was taken, and blood was collected for determination of ethanol concentration. In behavioral testing, a multiple-test testing battery can introduce carry-over effects, whereby the experience of one assay interferes with subsequent assays. The order of the behavioral tests in this battery was based upon previous work (Hamre et al., 2007) that demonstrated differential responses to ethanol or differences in ethanol consumption in three standard inbred strains of mice. The control mice underwent the same testing battery, enabling us to determine the effect of the knockout on phenotype.

#### Body temperature

Body temperature was recorded rectally using a digital thermometer (BIO-TK8851, EB Instruments, Pinellas Park, FL) with a B Ret-3 probe designed for mice (L: 19 mm; diam: 1.8 mm; Cable: 100 cm). The difference between the pre-EtOH temperature and the post EtOH temperature was considered the hypothermic response. This approach was used on mice in both cohorts 1 and 2.

#### Accelerating rotarod

This approach was used on cohort 1, mice that receive a non-sedative dose (1.25 g/kg) of ethanol. For each trial, each mouse was placed in one chamber of the rotarod facing the back wall of the apparatus. The rod was spinning at zero revolutions per minute (r.p.m.) at the beginning of the test and accelerated to 20 r.p.m at a rate of 1 rpm/6 s. The 3- in-diameter rod was covered with 320-grit automotive sandpaper (Rustay et al., 2003). The latency to fall was recorded. On day 1, each mouse was trained on 10 trials on the rotarod. Because the latency to fall generally stabilizes after five trials, the last three of the 10 trials were used to calculate the mean latency to fall. On day 2, for analysis of ethanol-induced ataxia, only three trials were given and all were used to compute a per- mouse average post-ethanol latency.

#### Loss of righting reflex (LORR)

This assay was performed only the second cohort of mice. This group received a sedative dose of EtOH (2.5 g/kg), and were assayed for hypothermia as well as LORR measured. The LORR test is used to measure the hypnotic/sedative effects of acute ethanol and produces two time measures: latency to loss of righting reflex and duration of loss of righting reflex (DLRR). Based on the original protocol (Crabbe et al., 1981) modified for increased sensitivity (Ponomarev and Crabbe, 2002), mice were weighed and then injected i.p with ethanol (2.5 g/kg, 20% EtOH v/v). Mice were placed in a cylindrical restrainer in an upright position (Stoeltinger Co., Wood Dale, IL). The restrainer is a hollow cylinder attached to a square base at one end and an adjustable lid/moveable disk at the other open end that is used to position the head. Once a mouse was placed in the restrainer, the horizontal restrainer was rolled 90 degrees to the right every 2–3 s. For the first few iterations of this procedure, prior to the onset of the ethanol's effect, the mice immediately right themselves. After 10–20 of such tests, the mice tend to remain on their back (for ~5 s) after two successive 90-degree turns. The LORR was calculated as the time interval between the onset of injection and the end of the second successive failed righting attempt. The DLRR is recorded as the time between injection and return of the righting reflex. This was measured in 3–10-min intervals following i.p injection. Return of the righting reflex occurs if animals can right themselves from a supine position within a 5-s period or could not be placed on their back after eight successive 90-degrees turns in the restrainer.

#### Locomotor activity in the open field

The open-field arena was an opaque Plexiglas box (39 × 39 × 39 cm) with a dark gray floor, illuminated at 43 ± 4 lux in a 10 × 15 feet testing room. A 10 × 10 cm center zone was defined for analysis. Each mouse was placed into the center of the arena and allowed to explore for 20 min. Behavioral measures in the open field and light-dark box were recorded, including fecal boli in the open field and light-dark box and analyzed by real-time video tracking using Ethovision XT 8. The distance traveled in the first 4 min (locomotor activity response to novelty); total distance traveled (general locomotor activity); percentage of time in the center; and defecation/number of fecal-boli (anxiety-like behaviors) were obtained for each mouse before and after alcohol injection.

#### Light-dark box

The light-dark box comprised a box with an insert evenly dividing the open-field apparatus into light-dark compartments, with the light compartment illuminated at 17 ± 2 lux. Mice were placed into the dark compartment and a 20-min recording began when the lid was closed (Henderson et al., 2004). The following behaviors were measured percentage of time spent in the dark.

#### Blood ethanol concentration (BEC)

At the completion of alcohol-effect testing of cohort 1, 1 h following ethanol injection, 30 μl of serum was obtained by retro-orbital bleeding. BEC was analyzed on a Beckman DXC clinical system.

#### Alcohol preference testing

Two-bottle free-choice methods were followed as previously described (Bachmanov et al., 2002; Mulligan et al., 2008). Individually housed mice from cohort 2 were presented with two tubes containing an ethanol solution (VWR International Radnor, PA) made in sterilized acidified (pH 2.5–3) water or sterilized acidified water alone. The positions of the tubes were switched daily to avoid side preference effects. Sipper tubes were weighed daily in the middle of the light cycle, to the nearest 0.01 g to measure the consumption of each solution. For 8 days prior to testing, mice were habituated to single housing with multiple sipper tubes, each containing sterilized acidified water. The experimental period occurred over 20 days, during the first 4 days of which the mice received 3% ethanol (v/v) and during the next 4 days of which they received 6% ethanol, followed by 4 days of each 9, 12, and 15% ethanol. Control bottles of ethanol and water were placed in an empty mouse box, weighed daily and their positions alternated. This weight was subtracted from daily results to account for loss in experimental samples due to handling and evaporation. The weight of the mouse after the protocol was used to calculate ethanol consumed and total consumption per kg body weight, there was weight data missing from six mice, which were excluded from these calculations but included in preference.

### Statistical analysis

All analyses of mutant experiments were conducted using JMP 10 (SAS Institute). Alcohol preference was calculated as the amount of alcohol consumed as a percentage of the total alcohol and water consumed for both 3% and 10% EtOH. The full model is

$$\begin{array}{l} \text{Preference}=\beta_{0}\text{Sex}+\beta_{1}\text{Genotype}+\beta_{2}\left(\text{Sex}\times \text{Genotype}\right)+\varepsilon , \end{array}$$

where ε is random error. The parameters (β, ε) were estimated by Type III non-sequential ordinary least squares in the ANOVA model. Repeated measures analysis of variance was performed using MANOVA to estimate the effects of genotype, sex and their interaction on both pre- and post-treatment measures. In all cases, the full model was fit and reduced by dropping non-significant interactions followed by main effects.

For ethanol-induced hypothermia,

$$\begin{array}{l} \text{Temperature}=\beta_{0}\text{Sex}+\beta_{1}\text{Genotype}+\beta_{2}\left(\text{Sex}\times \text{Genotype}\right)\\ \;+Ṵ+\varepsilon , \end{array}$$

where ε is random error. The parameters (β, ε) were estimated by Type III non-sequential ordinary least squares in the ANOVA model. Repeated measures analysis of variance was performed using MANOVA to estimate the effects of genotype, sex and their interaction. Ṵ represents the symmetric nature of the variance co-variance matrix of the random effects. In order to determine the nature of differences detected in the ANOVA model, planned contrasts were performed giving the terms not included in the model a weight of zero and giving the terms to be compared (sex and genotype) values of opposite weights, −1 and +1.

To adjust for the multiple testing of mice tested in cohorts 1 and 2, a false-discovery rate *q*-value was calculated using the R package qvalue, setting fdr. level = 0.05, pi0.method = “bootstrap,” adj = 1.2 (Storey and Tibshirani, 2003). Significant values were those with a familywise alpha < 0.05.

## Results

### Integrative functional genomic analyses

**Figure 1:**
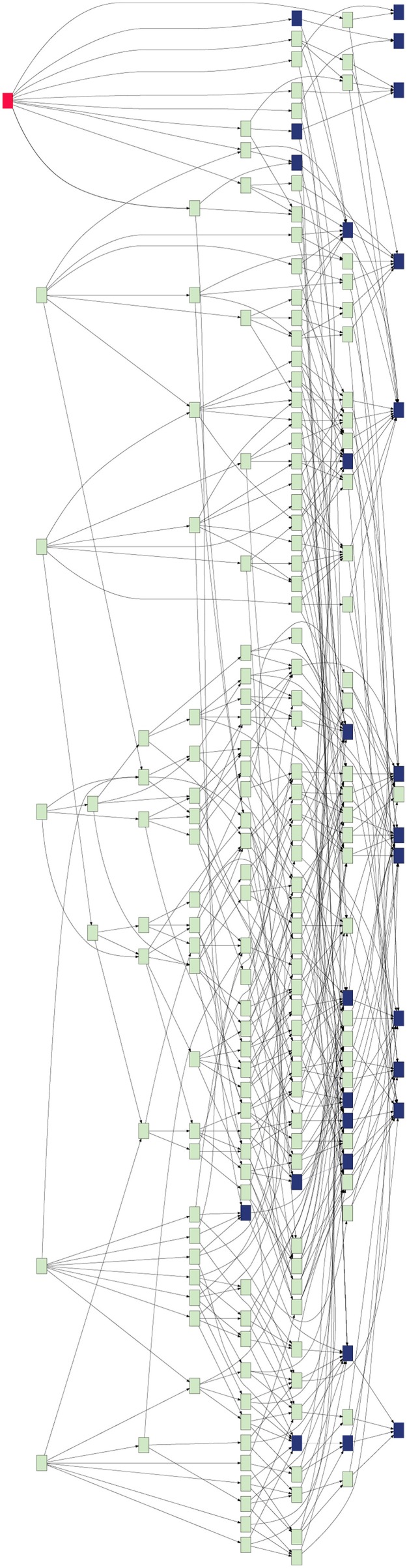
**Hierarchical similarity graph of experimentally derived alcohol-related genes sets**. Terminal nodes, which are shown at the right-most level of the graph, represent the individual gene sets retrieved from the GeneWeaver database and each level above reflects higher order intersections of these sets, such that the highest order intersections are represented at the left of the graph. Each node represents a biclique, that is, a set of genes completely connected to a group of gene sets. The most highly connected gene is *Pafah1b1* (red). Nodes representing bicliques that contain known alcohol-related genes from OMIM and MP annotations are represented by blue shading. Note that the majority of known alcohol-related genes are each found on only a small number of the experimentally derived gene sets, and do not populate the highly intersecting experimental results, whereas many genes not previously associated with alcoholism are found in a greater number of gene set intersections.

GeneWeaver's database was queried for alcohol-related gene sets. The analysis was restricted to experimentally derived gene associations from genome-wide experiments across three major data types (differential expression, gene expression-phenotype correlations, and QTL positional candidates), which resulted in 32 gene sets (Table S1) from five species (*Mus musculus, Rattus novregicus, Danio rerio, Drosophilia melanogaster*, and *Homo sapiens*). GeneWeaver's Hierarchical Similarity Graph tool was used to perform a *de novo* aggregation of these 32 datasets based on the genes they contain (Figure 1). GeneWeaver represents the result as a bipartite graph. In such a graph, there are two disjoint sets of vertices, or nodes, with genes represented in one partite set, and gene set identifiers represented in the other. Genes and gene set identifiers (vertices) are connected by edges to represent gene set membership. The sets were harmonized to a common unique identifier for each cluster of gene identifiers defined by homology mapping. The union of all gene sets represented 13,609 genes across five species, with 6647 unique clusters of homologous genes, connected to gene sets by 10,684 edges. Briefly, this analysis enumerates all set-set intersections and represents them in a hierarchical graph (Figure 1) of the relations among the intersections, such that individual gene sets are represented at the right of the graph and successively higher-order intersections at the left. Each node in the hierarchical similarity graph represents a set of genes completely connected to a group of input gene-sets (Baker et al., 2009). This completely connected sub-graph of the bipartite graph is referred to as a biclique. The proportion of populated nodes gives an estimate of the cohesiveness of experimental results associated with a given concept. Fewer, larger combinations of gene sets, indicate similarity of experimental results related to the search term, whereas a greater number of small combinations indicate fragmentation of the results, and a less cohesive search term. Of the 8.5 billion possible intersections, 2297 of these were populated in the Hierarchical Similarity Graph, including 456 nodes representing intersections populated with more than two genes. To prune the Hierarchical Similarity graph we applied bootstrapping, which sampled 75% of gene set members and generated graphs for 1000 iterations. Only nodes and edges found in more than 50% of bootstraps were retained. The resulting bootstrapped graph (Figure 1, Figure S1) represents these robust gene set intersections and contains 195 intersection nodes and 543 edges.

**Figure 2:**
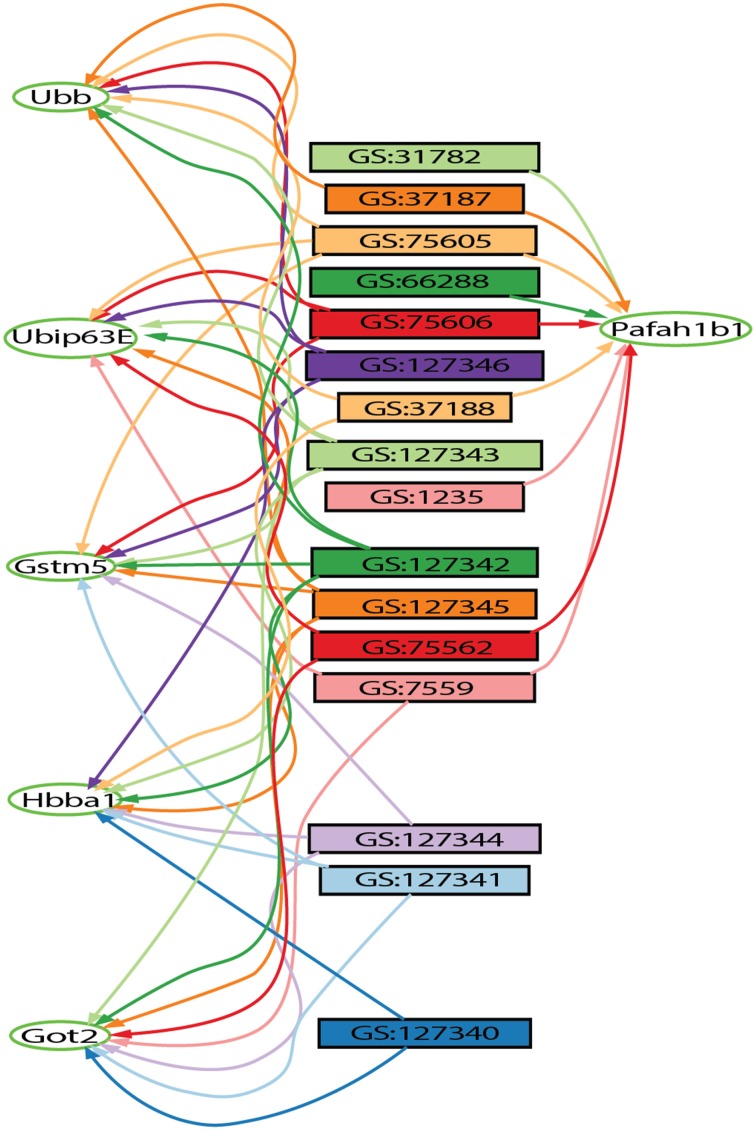
**Output from the gene set graph tool showing *Pafah1b1*-connected gene sets and other high-degree genes found on these sets**. The genes are represented by oval shaped nodes, edges represent gene set membership, and the rectangular nodes represent gene sets retrieved from the GeneWeaver database. Other highly connected genes include *Gstm5, Ubb, Hbaa1, Got2*, and *Ubi-p63E*.

The Hierarchical Similarity Graph was then highlighted using a derived gene set of known alcohol related genes. This set includes genes that had either been annotated to an alcohol related MP terms (Smith and Eppig, 2009), or annotated in the Online Mendelian Inheritance in Man (OMIM) database (Amberger et al., 2015) to alcohol-use disorders. This analysis reveals that although many genes have been identified in individual studies of alcoholism, the most highly represented genes among the 32 gene sets are not currently annotated to alcoholism. The intersections containing these genes were at most only four levels above the terminal nodes of the hierarchical similarity graph. The left five levels of the hierarchical similarity graph contained no genes previously annotated to alcohol or alcoholism. The left two levels, a total of six nodes, represent six distinct genes currently not associated with alcohol in single gene studies, but found at the intersection of multiple genome-wide experiments. The Hierarchical Similarity Graph comprises a data-driven organization of experiments with different regions potentially representing categorically different subsets of experimental results. Examination of the gene sets associated with the most highly connected genes (Figure S1), reveals regions or subspaces of the graph that represent species separation, for example, experiments in fly and human implicate genes not found in the overlap of mouse and fly experiments. Another observation is that there are regions of the graph that represent overlap of gene sets from the same experimental study. The gene in the highest level node of the hierarchical similarity graph was *Pafah1b1* (Figure 2), platelet-activating factor acetyl-hydrolase, isoform 1b, subunit 1, also known as *Lis-1*, for lissencephaly, type 1. This gene was present among 28% of sets, representing data from 4 of the 5 species included in the analysis (Table S2).

### Functional validation of a role for *Pafah1b1* in alcohol response in mice

The laboratory mouse is a powerful and efficient model system that has been used in many studies of alcohol drinking and alcohol response (Crabbe, 2014). Furthermore, previous work has shown strong conservation of *Pafah1b1* in mouse and human (Hirotsune et al., 1998). *Pafah1b1* has been shown to be co-expressed with GABA type-A receptor subunits (specifically *Gabra1, Gabrb2*, and *Gabrg2*), a family of neurochemical receptors associated with alcohol-use disorder (Mulligan et al., 2012). Hence, using mice to validate the role of *Pafah1b1* in alcohol response is indicated. Mutations in the human gene PAFAH1B1 result in neuronal migration defects including lissencephaly, a smoothened cerebral cortex (Lo Nigro et al., 1997). Because several gene sets from the GeneWeaver analysis indicated differential regulation of this transcript in response to alcohol (Lewohl et al., 2000; Mulligan et al., 2011), we tested the hypothesis that perturbation of this gene affects acute behavioral and physiological response to alcohol. Other studies revealed increased *Pafah1b1* abundance in mice selected for increased alcohol drinking so we also studied the preference for alcohol.

### Effects of heterozygous *Pafah1b1* deletion on alcohol response

As homozygous deficiency of *Pafah1b1* results in embryonic lethality (Tokuoka et al., 2003), we tested heterozygous deficient mice (129S-*Pafah1b1*^*tm*2.2*Awb*^/Ejc) and compared them to their wild-type (WT) litter mate controls. In the present study we tested two cohorts of mice for alcohol response in two batteries of tests (Table S3). The first cohort received a low dose of ethanol, 1.25 g/kg and the second cohort received two higher sedative doses of ethanol, 2.25 and 2.5 g/kg at different ages. Cohort 1 comprised 18 heterozygous mutants (eight females and 10 males) and 22 WT mice (11 females and 11 males) at 18 weeks of age, and cohort 2 comprised 16 heterozygous mutants (10 females and six males) and 13 WT mice (10 females and three males) tested at both 18 weeks of age and again at 6–8 months of age (sufficiently removed from initial testing to avoid development of chronic tolerance). Cohort 1 was evaluated pre- and post-ethanol exposure using behavioral tests of locomotor activity in an open field, locomotor response on an accelerating rotarod, locomotor activity in a light-dark box and hypothermia. Cohort 2 was evaluated pre- and post-ethanol for hypothermia and loss-of-righting reflex. Cohort 2 was then tested for alcohol preference over an increasing series of ethanol concentrations (3, 6, 9, 12, and 15% v/v EtOH). Below are the results of the various tests.

#### Hypothermia

**Figure 3:**
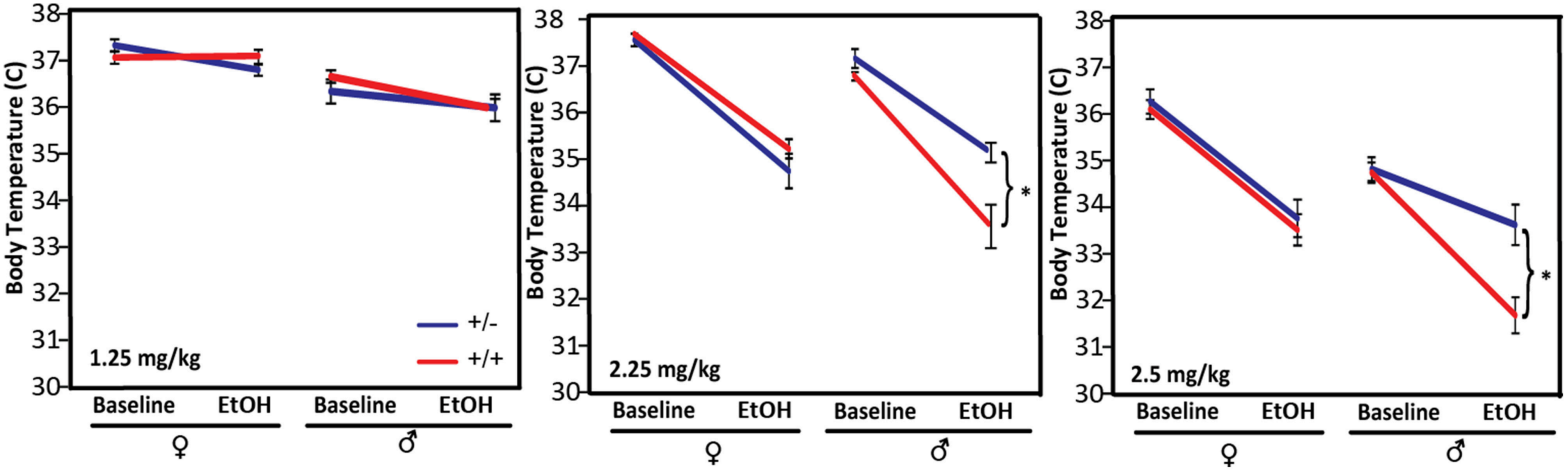
**Hypothermic response to different doses of ethanol, 1.25, 2.25, and 2.5 g/kg**. Means ± SEM are depicted for 1.25 g/kg (8 ± ♀, 10 ± ♂, 11 +∕+ ♀, 11 +∕+ ♂), 2.25, and 2.5 g/kg (10 ± ♀, 6 ± ♂, 10 +∕+ ♀, 3 +∕+♂). ^*^p < 0.05.

The magnitude of and tolerance to hypothermia in response to alcohol administration has been shown to be negatively correlated with withdrawal severity and is used as a predictive marker for susceptibility to ethanol dependence in genetic studies of rodents (Crabbe et al., 1983). We tested the hypothermic response of *Pafah1b1* mutant mice to three different doses of alcohol: in cohort 1, 1.25 g/kg, and in cohort 2, 2.25 g/kg and 6–8 weeks later, 2.5 g/kg (Figure 3). Compared to WT male mice, *Pafah1b1* mutant mice have a significantly decreased hypothermic response to 2.25 and 2.5 g/kg alcohol but not to 1.25 g/kg EtOH. There was a significant sex × genotype interaction [*F*_(2, 23)_ = 18.2494, *p* < 0.0001, *q* < 0.0001] independent of dose (2.25 and 2.5 g/kg were repeated measures of the same mice). At 2.25 g/kg, the body temperature of WT males decreased by 3.16 ± SEM = 0.41 degrees, whereas the body temperature of the *Pafah1b1* mutant males decreased by only 1.98°C ± SEM = 0.10 degrees. The 2.5 g/kg dose followed the same pattern. At 1.25 g/kg, the dose used in cohort 1, there was only a univariate sex and ethanol treatment effect in all mice [*F*_(1, 38)_ = 44.0213, *p* < 0.0001, *q* = 0.024; *F*_(1, 38)_ = 6.9, *p* = 0.0119, *q* = 0.034]. These results suggest that the heterozygous deficiency of *Pafah1b1* in males results in an attenuated hypothermic response to higher (sedative) doses of ethanol.

#### Ataxia-motor incoordination

**Figure 4:**
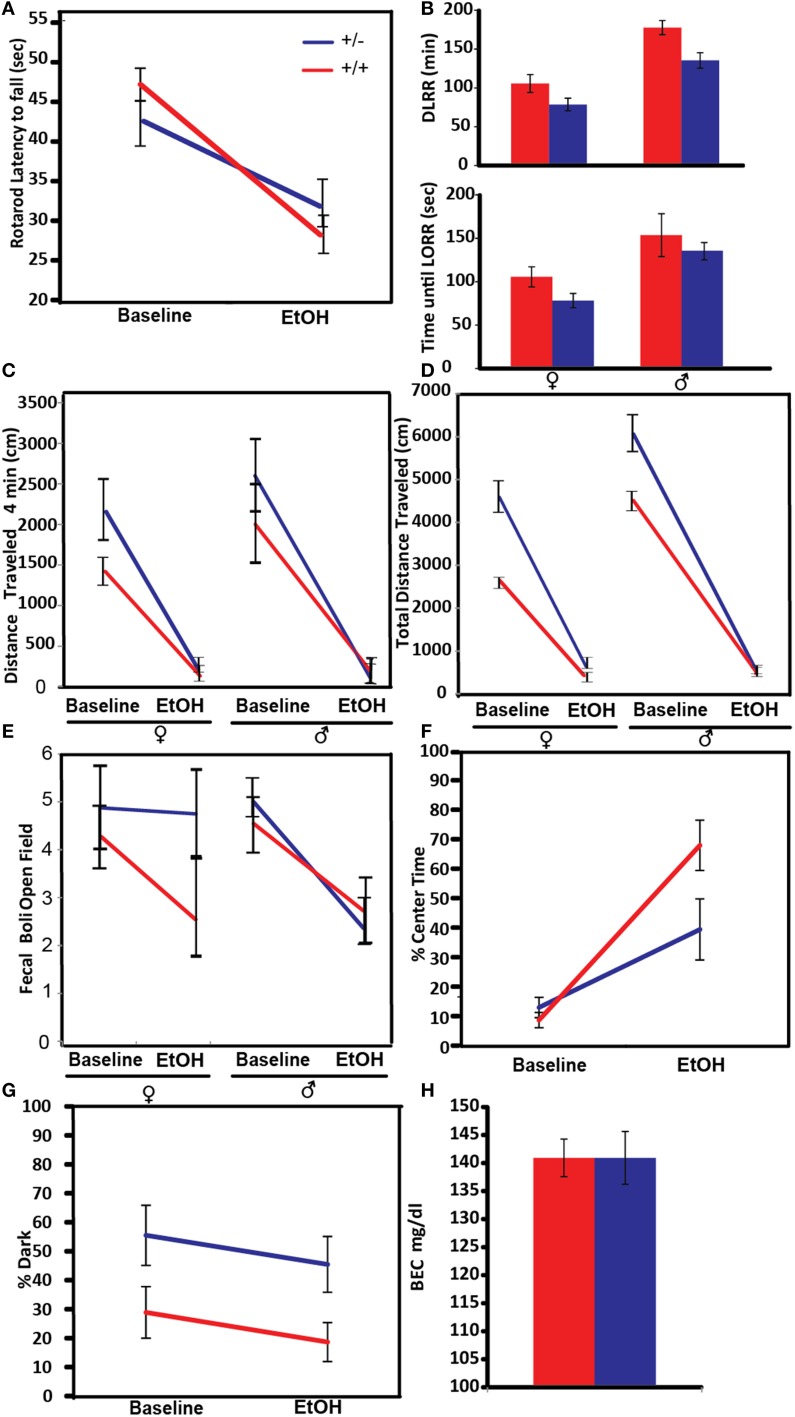
**Effects of *Pafah1b1* heterozygous knock-out on the acute effects of alcohol on different behavioral and physiological traits**. For each figure, red indicates WT mice and blue indicates *Pafah1b1* heterozygous mutant mice. **(A)** Ataxia was measured via latency to fall from an accelerating rotarod before and after a 1.25 g/kg EtOH. **(B)** Sedation measured in response to 2.5 g/kg EtOH as measured by time until LORR and time to recovery from LORR (DLRR). **(C)** Distance traveled after the first 4 min in a novel environment. **(D)** Total distance traveled in a novel environment. **(E)** Anxiety as measured by counts of fecal boli in the open field after 20 min. **(F)** Percentage of time in the center of the open field as a measure of anxiety. **(G)** Percentage of time in the dark as a measure of anxiety. **(H)** Blood ethanol concentration 1 h following 1.25 g/kg dose of EtOH. Means ± SEM are depicted, +/+ *n* = 22, ± *n* = 18, except **(B)** (cohort 2, +/+ *n* = 13, ± *n* = 16).

An increased overall sensitivity to acute alcohol may be protective against alcohol-use disorders (Schuckit, 1994). In this experiment ataxia, as a measure of alcohol sensitivity, was tested by measuring the latency to fall from a spinning rotarod (Figure 4A). Both control and *Pafah1b1* mutant animals exhibited alcohol-induced ataxia on the rotarod at 1.25 g/kg EtOH. There was a significant genotype × treatment interaction manner with crossover effects [*F*_(1, 38)_ = 5.9395, *p* = 0.0196, *q* = 0.041] such that *Pafah1b1* mutant mice were less coordinated than WT mice at baseline but were more coordinated than WT mice after alcohol exposure.

#### Sedation-loss of righting reflex (LORR) and duration of loss of righting reflex (DLRR)

Sensitivity to alcohol-induced sedation was tested by determining the time until the mouse lost its ability to right itself in a rotating chamber and the duration of time until this reflex was regained. *Pafah1b1* mutant and litter-mate control mice from cohort 2 were given a sedative dose 2.5 g/kg of ethanol (Figure 4B). There was a significant effect of sex and genotype [*F*_(2, 23)_ = 11.5531, *p* = 0.0004, *q* = 0.0001]. There was no interaction of these factors on time to LORR in response to alcohol injection. Both *Pafah1b1* mutant males and females had a lower LORR compared to WT mice, and females had a more rapid LORR than males. Similar effects were observed with time to recovery (DLRR). There was a significant effect of sex and genotype, but no interaction [*F*_(2, 22)_ = 16.3215, *p* < 0.0001, *q* = 0.0001]. The *Pafah1b1* mutant mice recovered more quickly than the WT, and the females recovered more quickly than the males.

### Anxiety

Prior research has shown a high rate of co-occurrence of alcohol-use disorders and anxiety disorders (Alegria et al., 2010). It is thought that the significant anxiolytic effects of alcohol in patients with basal elevated anxiety may promote greater alcohol intake, which can lead to abuse (Robinson et al., 2009). We evaluated measures of anxiety-like behavior in *Pafah1b1* mutant mice using measures of activity in a novel environment, defecation in the open field, percent time in the center of an open field and amount of time in the dark in a light-dark box.

#### Locomotor activity in the open field

Anxiety-like behavior in the open field is often assessed by measuring exploratory behavior within the first few minutes of beginning the open field test (Prut and Belzung, 2003), an approach that we took in this experiment. The open field assay was done at baseline in a novel environment and the following day in response to 1.25 g/kg EtOH. The distance traveled in the first 4 min (Figure 4C) showed a genotype × treatment effect [*F*_(1, 36)_ = 6.31, *p* = 0.016, *q* = 0.041], where the *Pafah1b1* mutants of both sexes traveled a greater distance compared to the WT mice. Total distance traveled in the open field (Figure 4D) showed a non-significant main effect of sex × treatment [*F*_(1, 37)_ = 4.46, *p* = 0.0415, *q* = 0.441] and a significant univariate genotype effect [*F*_(1, 37)_ = 4.5665, *p* = 0.0393, *q* = 0.046] where the males traveled a further distance and the distance was much greater in *Pafah1b1* mutants than in controls (5176 cm ± SEM = 945 vs. 3432 ± SEM = 336).

#### Defecation in the open field

The amount of defecation, as measured by the number of fecal boli in the open field at the conclusion of the open fie (Storey and Tibshirani, 2003) ld assay is used as an assay of anxiety. The fecal boli in the open field test results (Figure 4E) showed an anxiolytic effect of 1.25 g/kg EtOH [*F*_(1, 36)_ = 14.2132, *p* = 0.0006, *q* = 0.024] with a non-significant sex × treatment interaction [*F*_(1, 36)_ = 4.33502, *p* = 0.0442, *q* = 0.441] where female mice had a smaller change in response to ethanol, than the males. Genotype had no significant effect on the fecal boli response to alcohol [*F*_(1, 36)_ = 2.3450, *p* = 0.1344, *q* = 0.063].

#### Open field center time

We also tested anxiety via the percentage of time spent in the center of the open field box (Figure 4F). The percentage of time increased in response to 1.25 g/kg EtOH treatment [*F*_(1, 38)_ = 34.0289, *p* < 0.0001, *q* = 0.024] with a significant genotype × treatment interaction [*F*_(1, 38)_ = 4.9928, *p* = 0.0314, *q* = 0.044], where *Pafah1b1* mutants increased their center time in response to alcohol to a lesser degree than the litter-mate controls.

#### Light dark preference

The percent time spent in the dark compartment of the light-dark box was used as a measure of anxiety to the aversive light side (Figure 4G). In contrast to the observed increased exploratory activity in the open field, in the light-dark box mutant mice at baseline and post-ethanol injection tended to avoid the light to a greater extent (~26% increase) compared to control mice, suggesting increased anxiety. The percentage of time in the dark showed a genotype effect [*F*_(1, 37)_ = 6.3455, *p* = 0.0162, *q* = 0.041] but failed to show a treatment effect [*F*_(1, 37)_ = 1.8333, *p* = 0.1840], as the percent time in the dark post-ethanol was not different from baseline. These results suggest the 1.25 g/kg dose of EtOH was not sufficient enough to reduce the anxiety produced by the light side in either strain of mice.

#### Blood ethanol concentrations

The concentration of ethanol in the blood 1 h after injection with 1.25 g/kg was not significantly different between the *Pafah1b1* mutants and WT mice [*F*_(1, 39)_ = 0.0105, *p* = 0.9191, 140.92 mg/dl ± SEM = 4.70 vs. 141.50 mg/dl ± SEM = 3.35; Figure 4H].

### Effects of heterozygous deletion of Pafah1b1 on alcohol preference

#### Alcohol preference

**Figure 5:**
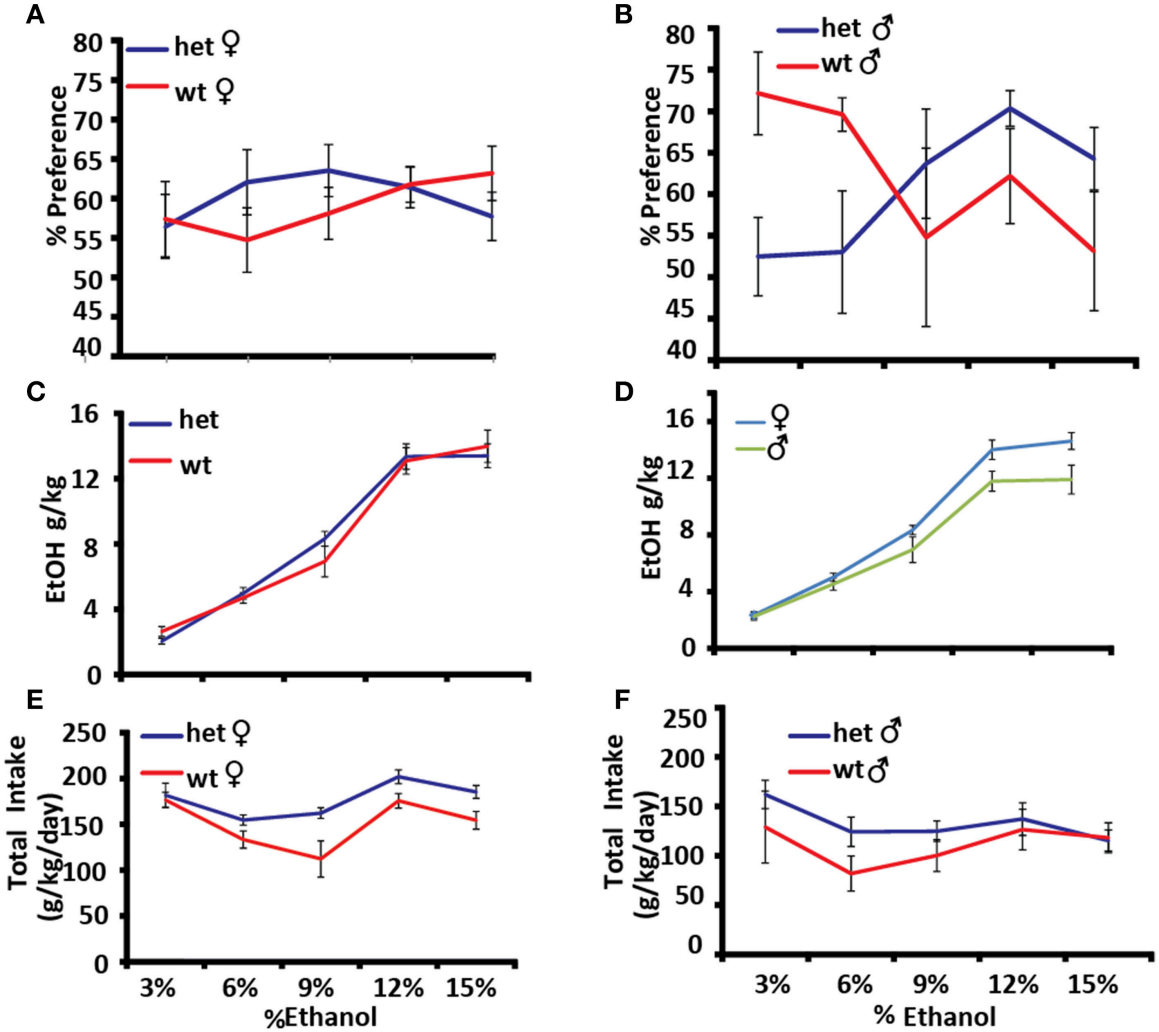
**Effects of *Pafah1b1* heterozygous knock-out on alcohol preference. Alcohol preference was measured using the two-bottle choice paradigm for 4 days at increasing concentrations of ethanol (3, 6, 9, 12, and 15%). (A)** Alcohol preference expressed as a percentage of alcohol consumed divided by total fluid intake in females. **(B)** Alcohol preference expressed as a percentage of alcohol consumed divided by total fluid intake in males. **(C)** Alcohol consumed as g/kg in *Pafah1b1* mutants and WT controls. **(D)** Alcohol consumed as g/kg in males and females. **(E)** Total fluid consumed as g/kg/day females. **(F)** Total fluid consumed as g/kg/day males. Means ± SEM are depicted **(A,B)** +/+ *n* = 13, ± *n* = 16, male and female **(C–F)** +/+ *n* = 10, ± *n* = 13, male and female.

Alcohol preference was measured using the two-bottle choice paradigm, in which one of two adjacent bottles contains ethanol and the other contains water. The experiment was carried out for 4 days at increasing concentrations of ethanol [3, 6, 9, 12, and 15% (v/v)] (Figures 5A–F). Genotype × sex × dose had a significant effect on alcohol preference as expressed as a percentage of total fluid consumed [*F*_(4, 22)_ = 3.2374, *p* = 0.0312]. At low doses male *Pafah1b1* mutant mice avoid ethanol compared to WT controls and prefer it at higher doses, whereas *Pafah1b1* female mice trend toward preferring ethanol across all doses (Figures 5A,B). Alcohol intake in the two-bottle choice paradigm shows a trend toward a significant genotype × dose effect [*F*_(4, 15)_ = 2.8029, *p* = 0.064] with the *Pafah1b1* mutants drinking more ethanol than the WT (Figure 5C) and a significant univariate sex effect [*F*_(1, 18)_ = 5.9240, *p* = 0.0256] where females drink more alcohol overall (Figure 5D). Total consumption of fluids over increasing doses shows a genotype × sex × dose interaction [*F*_(4, 16)_ = 3.5023, *p* = 0.0309], where female *Pafah1b1* mutants drank the most ethanol over all doses and the wild type males drank the least (Figures 5E,F).

## Discussion

Through integration and analysis of genome-wide alcohol response experiments performed across species, curated from disparate sources and integrated in the interactive computational framework (GeneWeaver), multiple previously unknown alcohol related genes were identified, one of which *Pafah1b1* was validated experimentally. Many others remain to be evaluated but are prioritized through convergence of evidence from among thousands of individual candidates. This integrative genomics analysis of diverse experimental data revealed a potential novel role for *Pafah1b1*, a gene that had not been previously associated with alcohol-use disorder. This finding was confirmed using a knock-out mouse. The striking finding highlighted in Figure 1 is that the most highly connected genes, i.e., the genes present in the highest number of datasets from the alcohol-related studies analyzed in GeneWeaver, were not previously annotated to alcohol response in humans, as indicated in OMIM, or in mice, as indicated in the MP Ontology. Although we were surprised to find this, it is possible that the sparse annotation in OMIM and MP for the genes we identified from experimental data is the result of their essential roles in development, as in such cases a complete null would be lethal, as is the case with *Pafah1b1* (Georgi et al., 2013). It is likely that the historical stringency of reporting for genome-wide experimental studies has been too high, resulting in a large number of false negative results that have escaped attention in initial analyses. The GeneWeaver system, and its underlying algorithms for gene set integration, addresses this issue by harmonizing diverse data, thereby reducing sparsity, and enabling integrative analysis to find convergent evidence for genes which may have weak but frequent experimental evidence for a role in disease. GeneWeaver captures and distills the experimental designs, analysis approaches, curation methods, and decision criteria imposed by experimenters to associate genes to biological concepts into a homogeneous data structure. We can represent the relations of biological entities, in this case genes, and the experimental constructs to which they are associated.

Mouse mutant models are a powerful system in which to evaluate the roles of genes in alcohol-related disorders. Several of the characteristics of alcohol-use disorder can be modeled directly or indirectly in mice, including alcohol preference, tolerance, and withdrawal (Crabbe, 2012). Alcohol preference as measured in a two-bottle choice paradigm is highly heritable, and is inversely correlated to withdrawal severity in rodents (Ford et al., 2011). The magnitude of and tolerance to hypothermia in response to alcohol administration has been shown to be negatively correlated with withdrawal severity and is used as a predictive marker for susceptibility to ethanol dependence in genetic studies of rodents (Crabbe et al., 1983). In the heterozygous *Pafah1b1* mutant, we observed increased hypothermia in a dose-dependent manner in males (Figure 3) and a decreased preference for ethanol at low doses in the male mutants (Figure 5B). The female mutants showed no difference in hypothermic response (Figure 3) but exhibited a trend toward increased preference (Figure 5A). *Pafah1b1* heterozygous mice showed increased basal anxiety (percentage of time in the dark at baseline) as well as hyperactivity. There was less response to alcohol observed in the center of the open field in the *Pafah1b1* mutant mice (Figure 4F). *Pafah1b1* heterozygous deficiency results in decreased anxiolysis in response to ethanol. The increased basal activity (hyperactivity) of the mutant mice in the open field, however, was restored to WT levels in response to ethanol, indicating that the 1.25 g/ml EtOH was sufficiently able to counteract the *Pafah1b1* mutant's baseline hyperactivity. Both the sex and the genotype had effects on alcohol preference across the dose series. The most profound difference was the reduced preference for 3% seen in the male *Pafah1b1* mutants that then crossed over at the higher doses (12–15%) and became an increased preference. Overall, the female mutants also had slightly increased preference over WT across all doses. *Pafah1b1* mutation had a strong impact on the sedative effects of alcohol. Compared to the wild-type littermate controls the mutants of both sexes were sedated much more rapidly than the controls (Figure 4B) but also recovered much more rapidly than the controls. This suggests that the mutants are more sensitive to the effects of ethanol but are also able to recover from them more rapidly.

Although, we know of no previous targeted studies of the role of this gene in alcoholism, such a role is plausible. The underlying mechanisms of alcohol dependence involve multiple signaling pathways and substantial neuroadaptive changes (Cui et al., 2013; Mahoney and Olmstead, 2013), which are first engaged during the acute response to alcohol. These acute responses such as hypothermia, LORR, and anxiolysis were impacted by a heterozygous *Pafah1b1* mutant. The mechanism of *Pafah1b1's* action in alcohol response could function at several levels. First, *Pafah1b1* has a role in synaptic integration (Hunt et al., 2012). *Pafah1b1* mutations result in cortical, hippocampal and olfactory disorganization, reduced dendritic spine plasticity, impaired spatial learning, and coordination and social interactions (Paylor et al., 1999; Sudarov et al., 2013). The effects of alcohol on synaptic plasticity and neuromodulation are well-documented (reviewed in McCool, 2011). Second, *Pafah1b1* plays a role in excitatory neurotransmission facilitated by dynamin (Sudarov et al., 2013). Most of the excitatory synaptic transmission in the nervous system is mediated by N-methyl-D-aspartate (NMDA) receptors. The inhibitory role of alcohol on the activity of the NMDA receptor channels has been extensively studied in numerous brain tissues (e.g., Yaka et al., 2003; Kolb et al., 2005) and variants of the receptor are associated with alcohol-use disorder (Kim et al., 2006; Pastor et al., 2009). Finally, *Pafah1b1* plays a critical role in neuronal migration and cerebellar function (Cahana et al., 2001). Perturbation of this gene causes neuronal migration defects through cytoskeletal disruption, and its role in neuronal migration and synaptic function in cerebellar granule cells (Hunt et al., 2012) suggests a possible role for this gene in fetal alcohol syndrome (Jiang et al., 2008).

The scope of the literature on alcoholism, even restricted to the area of functional genomics, is vast. Through the use of integrated evidence and gene prioritization in GeneWeaver, it is possible to identify and prioritize research on novel roles for genes that are strongly implicated in phenomena such as alcohol-use disorders. GeneWeaver's data integration strategy is extensible to virtually any biological entity types in addition to the genes and gene products, providing facile integration and aggregation of convergent evidence. Many other applications of gene set integration exist (Bubier et al., 2015).

The increased emphasis and policy support for genomic data sharing will greatly accelerate the discovery of gene function in disease. The challenge remains, however, to extract meaningful biological signal from these data through the development of usable platforms and algorithms for interpretation of harmonized experimental results (Costello et al., 2013). The biomedical literature and the digital realm of biological data comprise a vast, yet sparsely annotated, and disparate collection of genomic data repositories, publication supplements, and curated experimental results. Accessing and integrating these extensive resources requires data integration and scalable computation and standardization. Truly making sense of the findings requires knowledge-discovery systems amenable to interrogation by functional biologists. GeneWeaver brings together convergent biological results from divergent species to identify highly supported but undiscovered roles of genes in disease, and to identify model organism resources and experimental strategies rapidly that are appropriate for confirmatory studies.

## Author contributions

JB, EC Designed research, analyzed data. EB, ML, JJ, EC contributed analytic tools, JB, TW, EC performed research. JB, TW, JJ, ML, EB, and EC wrote the paper.

## Funding

This project was supported by NIH R01 AA 18776 to EC. We gratefully acknowledge The Jackson Laboratory Scientific Services supported by NIH P30 CA034196.

### Conflict of interest statement

The authors declare that the research was conducted in the absence of any commercial or financial relationships that could be construed as a potential conflict of interest.

## Supplementary material

Figure S1 **Hi-resolution, Hierarchical Similarity Graph of experimentally derived alcohol-related genes sets, with the nodes labeled to identify the gene sets they contain**.

Table S1 **The 32 gene set from genome wide functional genomic studies retrieved from GeneWeaver.org**.

Table S2 **GeneWeaver gene sets containing *Pafah1b1***.

Table S3 **Testing Paradigm for the two cohorts**.
