# Supplementary material for: Cross-Species Integrative Functional Genomics in GeneWeaver Reveals a Role for Pafah1b1 in Altered Response to Alcohol
Source: Front Behav Neurosci. 2016 Jan 21;10:1. doi: 10.3389/fnbeh.2016.00001 (PMC4720795; doi:10.3389/fnbeh.2016.00001)
Supplement: Table S2 — GeneWeaver gene sets containing Pafah1b1. [file TableS2.DOCX]

| **GeneSet ID** | **Species** | **Set Type** | **Reference** | **Gene Set Name** | **Number of Genes** |
| --- | --- | --- | --- | --- | --- |
| GS75562 | *D.melanogaster* | Differential expression | ([23](#_ENREF_23)) | Differentially expressed gene in alcohol sensitivity selected lines | 2280 |
| GS75559 | *D.melanogaster* | Differential expression | ([23](#_ENREF_23)) | Differential expression sex x alcohol interaction in alcohol sensitivity selected lines | 1277 |
| GS75606 | *H.sapiens* | Differential expression | ([48](#_ENREF_48)) | Frontal cortex gene expression in alcoholic vs nonalcoholic humans (male uncomplicated cases) | 147 |
| GS37187 | *M.musculus* | QTL Positional Candidates | ([49](#_ENREF_49)) | Positional candidate genes from QTLs mapped in a (B6 x FVB) F2 for alcohol 2-bottle choice excessive consumption | 554 |
| GS31782 | *M.musculus* | Differential expression | ([50](#_ENREF_50)) | Correlates of hippocampus Gene Expression with ethanol preference in BXD mice. | 415 |
| GS75605 | *H.sapiens* | Differential expression | ([48](#_ENREF_48)) | Frontal cortex gene expression in alcoholic vs nonalcoholic humans, | 147 |
| GS37188 | *M.musculus* | QTL Positional Candidates | ([49](#_ENREF_49)) | Positional candidate genes from QTLs mapped in a (B6 x FVB) F2 for drinking in the dark | 1654 |
| GS35872 | *M.musculus* | Differential expression | ([51](#_ENREF_51)) | Striatum gene expression correlates of elevated plus maze closed arm entries in BXD RI mice | 52 |
| GS1235 | *R.norvegicus* | Differential  Expression | ([52](#_ENREF_52)) | Differentially expressed in response to mGlu5 agonist. | 27 |

**Supporting Information Table 2. GeneWeaver gene sets containing *Pafah1b1*.**
