# Supplementary material for: Cross-Species Integrative Functional Genomics in GeneWeaver Reveals a Role for Pafah1b1 in Altered Response to Alcohol
Source: Front Behav Neurosci. 2016 Jan 21;10:1. doi: 10.3389/fnbeh.2016.00001 (PMC4720795; doi:10.3389/fnbeh.2016.00001)
Supplement: Table S3 — Testing Paradigm for the two cohorts. [file TableS3.DOCX]

**Supporting Information Table 3. Testing Paradigm**

**Cohort 1**

| Test Day | Test | Time post EtOH | Length of Test |
| --- | --- | --- | --- |
| 1 | Baseline Locomotor |  | 10 min |
| 1 | Baseline Rotarod |  | 10 trials |
| 1 | Baseline LD |  | 20 min |
| 1 | Baseline Core Body Temperature |  | 1 reading |
| 2 | Locomotor Activity | 10 | 10 min |
| 2 | Rotarod | 20 | 3 trials |
| 2 | LD Test | 35 | 10-20mn |
| 2 | Core Body Temperature | 60 | 1 reading |
| 2 | BEC | 60 | 1 blood draw |

**Cohort 2**

| Mouse Age (weeks) | Test | Time post EtOH | Length of Test |
| --- | --- | --- | --- |
| 18 | Baseline Core Body Temperature |  | 1 reading |
| 18 | i.p. ethanol injection (2.25 g/kg) |  | 1 injection |
| 18 | Core Body Temperature | 60 | 1 reading |
| 24-32 | Baseline Core Body Temperature |  | 1 reading |
| 24-32 | i.p. ethanol injection (2.25 g/kg) |  | 1 injection |
| 24-32 | LORR | variable | 1 time |
| 24-32 | Core Body Temperature | 60 | 1 reading |
| 24-32 | DLRR | variable | 1 time |
| 24-32 | Single house and Sipper tube Exposure |  | 7 days |
| 24-32 | 2-Bottle Choice 3-15% 4 days each dose. |  | 20 days |
