# Supplementary material for: Cross-Species Integrative Functional Genomics in GeneWeaver Reveals a Role for Pafah1b1 in Altered Response to Alcohol
Source: Front Behav Neurosci. 2016 Jan 21;10:1. doi: 10.3389/fnbeh.2016.00001 (PMC4720795; doi:10.3389/fnbeh.2016.00001)

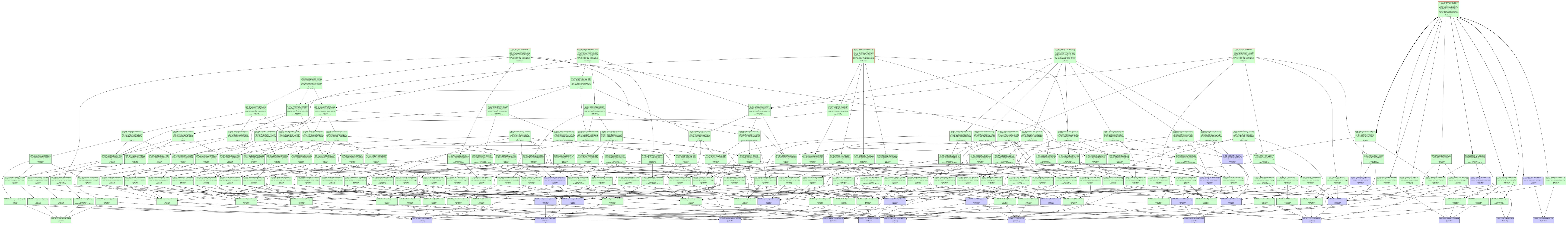

Supplement: Figure S1 — Hi-resolution, Hierarchical Similarity Graph of experimentally derived alcohol-related genes sets, with the nodes labeled to identify the gene sets they contain. [file FigureS1.PDF]
